# Structural Relationship Between Beef Food Quality, Trust, and Revisit Intention: The Moderating Role of Price Fairness Based on Heuristics Effect

**DOI:** 10.3390/nu17132155

**Published:** 2025-06-28

**Authors:** Kyung-A Sun, Joonho Moon

**Affiliations:** 1 Department of Tourism Management, Gachon University, Sungnam-si 13120, Republic of Korea; kasun@gachon.ac.kr; 2 Department of Tourism Administration, Kangwon National University, Chuncheon 24341, Republic of Korea

**Keywords:** beef product quality, price fairness, freshness, portion size, packaging, trust, revisit intention

## Abstract

Background/Objectives: Beef is a significant dietary component, and the concept of food quality is inherently complex and multidimensional. This study addresses this complexity within the context of beef products by examining four key attributes: price fairness, freshness, portion size, and packaging. It further investigates the structural relationships among food quality, consumer trust, and revisit intention, with particular attention to the moderating role of price fairness in the link between trust and revisit intention. Methods: Data were collected through an online survey administered via the Clickworker platform, targeting 415 U.S. consumers selected for their cultural relevance and substantial beef consumption. Structural equation modeling using maximum likelihood estimation was employed to test the hypotheses, and Hayes’ process model 15 was applied to assess the moderating effects. Results: The results show that consumer trust is positively influenced by all four quality attributes, and that freshness, portion size, packaging, and trust are positively associated with revisit and repurchase intentions. Moreover, price fairness demonstrated a significant moderating effect, framed within the heuristic decision-making perspective. Conclusions: This study contributes to the literature by shedding light on the determinants of consumer trust and repeat purchasing behavior in beef consumption. It underscores the heuristic role of price fairness and the interplay between perceived quality attributes and trust, offering practical insights for businesses seeking to enhance consumer perception and foster brand loyalty. However, this work is limited to the selection of the sample.

## 1. Introduction

**Figure 1:**
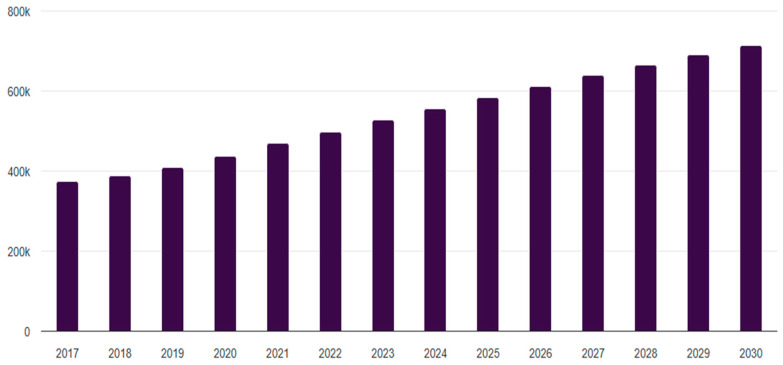
Global beef market size and forecasting. Available at https://www.grandviewresearch.com/horizon/outlook/beef-market-size/global, accessed on 24 October 2024.

Consumers purchase beef for various reasons, which might include its perceived health benefits and the enjoyment of flavorful food. However, the complexity of these motivations makes it challenging to identify which specific attributes most significantly influence beef purchasing decisions. Peri [1] highlighted the difficulty in defining food quality, as its interpretation varies across different contexts. Scholars have posited that consumers assess food products based on perceived quality [2,3,4], which ultimately drives purchasing behavior. Despite these insights, the existing literature has yet to adequately explore the definition of food quality within the specific context of beef products. This study aims to address this gap by investigating how consumers define and evaluate the quality of beef products. Additionally, this research focuses on the U.S. market, given its significant economic role in global beef consumption. According to Grand View Research [5], the U.S. beef consumption market was valued at USD 90.5 billion in 2022, representing approximately 18% of the global beef market, which was valued at USD 526.5 billion. Figure 1 shows that the global market size is forecasted as USD 712.5 billion by 2030. Furthermore, The Cattle Range [6] reported a consistent increase in U.S. beef consumption since 2015, with per capita consumption reaching 59.1 pounds in 2022. These figures suggest that U.S. consumers represent a key demographic for beef-related research. Given its substantial share in global beef consumption, the U.S. serves as an essential context for examining consumer behavior in the beef market.

This study identifies four key attributes of beef quality: price fairness, freshness, portion size, and packaging. The attributes used in this work were selected by considering aspects that are easy for consumers to verify when making a purchase. According to the European Commission [7], food quality is a multifaceted concept. The first domain is the price fairness. Previous research demonstrated that price significantly influences consumer decision-making [8,9]. The European Commission [7] also emphasized that price is a critical factor in evaluating food quality from the consumer’s perspective. The second piece of food quality is freshness. Freshness is included as a crucial sensory cue in food product evaluation [10,11]. Siroka and Strada [12] noted that the freshness of food is directly related to its sensory and nutritional value, indicating its importance in the overall assessment of food quality. Portion size is another key attribute, as it has been linked to positive outcomes such as reduced food waste and improved health [13,14]. Scholars argue that portion size, concerning eating habits, is a central dimension of food quality from the consumer’s perspective [15,16]. Lastly, packaging is considered an important attribute due to its role in product certification, presentation, and protection, all of which influence consumer perceptions [17,18]. Sohali et al. [19] and Yan et al. [20] highlighted that food packaging influences first impressions and product safety, suggesting that it serves as a key indicator of food quality in the marketplace.

The dependent variable in this study is revisit intention. Previous research has shown that repeated consumer visits contribute to increased sales for businesses, prompting numerous scholars to explore the factors that influence revisit intention [21,22,23]. In the context of beef, however, it is more appropriate to focus on purchasing from a specific location rather than repeat purchases as a more accurate indicator of consumer loyalty. Therefore, this study has selected revisit intention as the key attribute. This study incorporates the concept of trust, which has been widely explored in food market research for its role in fostering positive consumer behavior and enhancing sales growth [24,25,26]. Trust is hypothesized to influence revisit intention, the dependent variable in this study. Beef consumption occurs in various settings, such as butcher shops and supermarkets [27,28], making the context of place relevant for understanding consumer behavior. Furthermore, this research examines the influence of trust on revisit intention, a relationship well-documented in previous studies [29,30,31]. This study aims to confirm this association within the context of beef products.

This study investigates the moderating role of price fairness in two key relationships: between freshness and revisit intention, and between trust and revisit intention. Freshness is a critical factor in evaluating beef quality, but it can only be assessed visually, leading consumers to rely on various cues. In such cases, a low price may trigger skepticism about freshness, suggesting that discounting may not always be an effective marketing strategy for beef. While previous research has noted that price fluctuations can lead to consumer suspicion and negative reactions [32,33,34], little attention has been given to how this applies to premium products like beef. To address this gap, the present study examines how perceptions of price fairness moderate the effects of freshness and trust on consumers’ intention to revisit.

The primary objectives of this research are threefold. First, it aims to explore how food quality is defined in the beef market by examining four key attributes: price fairness, freshness, portion size, and packaging. Second, it seeks to analyze the structural relationships among beef quality attributes, consumer trust, and revisit intention. Third, the study investigates the moderating role of price fairness in the relationship between freshness, trust, and revisit intention, drawing on heuristics theory as the conceptual framework. Although a number of previous studies have proposed diverse perspectives on beef quality, empirical investigations focusing on consumer perceptions remain insufficient [35,36,37,38]. Furthermore, while the heuristic effect has been widely examined across various domains of consumer behavior [39,40,41], its application within the context of beef consumption has been largely overlooked. In this regard, the three objectives of the current study contribute meaningfully to the literature by addressing these critical research gaps. This research thus contributes to the literature by clarifying the definition of beef product quality and shedding light on consumer behavior in the beef market. Furthermore, it provides valuable insights for the sustainability of the beef industry by offering a better understanding of consumer preferences.

## 2. Literature Review and Hypotheses Development

### 2.1. Revisit Intention

Revisit intention refers to the desire of individuals to return to a specific location for commercial purposes, and is often considered an indicator of customer loyalty [21,22]. Researchers adopted revisit intention as a dependent variable because increased visitation typically leads to higher sales and, consequently, greater financial success for businesses [42,43]. As a result, prior studies have explored various attributes influencing revisit intention. For example, Han et al. [44] and Chun and Nyam-Ochir [45] investigated restaurant customers’ behavior, using revisit intention as a key variable. Al-Sulaiti [23] examined the determinants of revisit intention within the context of mega shopping malls. Similarly, Chang et al. [46] utilized revisit intention as a dependent variable to assess visitors’ perceptions of food festivals. Mandagi et al. [47] further explored the factors influencing revisit intention in hospital settings. A review of the literature reveals that revisit intention is widely used as a dependent variable across diverse domains.

### 2.2. Trust

Trust is defined as a firm belief in the reliability of particular goods in consumer behavior research [48,49]. Previous research has highlighted trust as a critical element, as it reflects a positive perception of products, which, in turn, influences consumers’ decision-making processes [25,50]. Wu and Huang [51] examined the antecedents of trust in the context of electronic commerce, while Min et al. [52] investigated consumer trust among retail consumers. Wu et al. [53] emphasized that trust arises from positive evaluations of food quality and safety. Given the crucial role of food in promoting individual health, Singh and Sharma [24] argued that trust, grounded in credible quality, is a central factor in the food industry. Murphy et al. [26] employed trust as a variable to explore consumer behavior in the organic food sector. Viktoria Rampl et al. [25] similarly examined consumer trust with a focus on food retailers. A review of the literature suggests that trust plays a significant role in understanding consumer behavior within the food industry.

### 2.3. Food Quality and Its Sub-Dimensions

Food quality is inherently complex and difficult to define, as scholars argue that it should be conceptualized by integrating various dimensions [54,55]. Previous literature noted that the definition of food quality is context-dependent, as consumer interests and preferences may vary across different markets [1,54]. Additionally, researchers contend that the complexity in defining food quality arises from the cultural context in which food is consumed [1,56,57]. Given these propositions, previous studies have sought to define food quality across diverse domains. For instance, Custodio et al. [58] defined rice quality, while Grunert et al. [59] examined the multidimensional nature of meat quality. Haile and Kang [60] explored food quality in the coffee industry, and Kamboj et al. [61] focused on organic food. Chowdhury [62] elaborated on the definition of food quality in the context of online business. A review of the literature suggests that food quality definitions are likely to differ depending on the specific context. Such a framework could be applied to the case of beef. To be specific, scholars addressed that beef quality could be defined using various attributes: healthiness, safety, sensory attributes, etc. [35,36]. Mwangi et al. [37] stated that nutritional value is an essential attribute to appraise the quality of beef. Liu et al. [38] presented that beef quality is defined as diverse attributes: appearance, price, nutrition, sensory attributes, etc.

Prior works also indicate that food quality is frequently assessed in terms of price, as consumers consider price to be a critical factor in their purchasing decisions [63,64]. As a staple necessity, food is primarily evaluated by its price, which plays a key role in consumer decision-making [8,9,64]. Several studies have utilized price fairness as an indicator of consumer perceptions regarding price levels, where price fairness refers to the rational evaluation of price with the perceived value of a product [65,66,67]. Previous research has shown that beef consumers, in particular, are highly price-sensitive due to the relatively high cost of beef compared to other food products [8,68]. Another important dimension of food quality is freshness, which refers to recently prepared food with optimal sensory qualities. Scholars claimed that freshness plays a significant role in food’s sensory appeal [10,11]. Since humans evaluate food using multiple senses, the freshness of a product is critical for positive consumer appraisal and decision-making [69,70,71]. In the case of beef, Lin et al. [72] noted that consumers often rely on sensory cues such as color and blood to assess freshness, influencing their purchasing decisions. Portion size is another essential attribute in the evaluation of food quality. It is defined as the amount of food consumed in a single eating occasion [15,16]. Portion size is linked to consumption amounts, and consumers typically prefer adequate portions, as excessive food consumption can lead to health problems such as obesity and diabetes [14,73,74]. Researchers also documented that food demand varies based on household size [13,75]. For example, Liu et al. [13] found that single-person households tend to require smaller portions, while larger households require greater quantities [13,76]. Accordingly, researchers argue that providing appropriate portion sizes is crucial for meeting consumer needs, as excessive portions can contribute to food waste [14,75]. Since beef consumption patterns vary based on household size and demographic factors such as gender and age, proper portion sizing is an important consideration from the consumer’s perspective [13,77]. Packaging, which refers to the method used to preserve products throughout the supply chain, is another significant attribute of food quality. Packaging serves both protective and aesthetic functions, contributing to the visual appeal and perceived quality of the product [78,79]. Packaging often includes certification labels, which reassure consumers about the quality of the product [80,81]. In the context of beef, previous studies have suggested that packaging not only protects the product but also conveys certifications that assure consumers of its quality, thereby influencing their purchasing decisions [17,18,82]. In summary, this research identifies four key attributes as sub-dimensions of beef quality: price fairness, freshness, portion size, and packaging.

### 2.4. Hypotheses Development

Previous works have consistently emphasized the critical role of food quality in consumer decision-making. For example, Nazri et al. [4] examined food truck consumers and found that food quality significantly enhanced repurchase intention. Similarly, Zhong and Moon [83] demonstrated a positive relationship between food quality and customer loyalty within the fast-food restaurant industry. Hidayat et al. [2] further confirmed a positive association between food quality and repurchase intention in the context of hot plate restaurants. In a related study, De Toni et al. [84] found that food quality positively influences consumer loyalty in the organic food sector. Halimi et al. [85] also reported a positive impact of food quality on revisit intention within the context of Muslim culture. Additionally, Temperini et al. [3] explored Italian consumers and discovered that food quality contributes to the development of higher levels of consumer trust. Price et al. [86] examined food consumers in workplace settings and highlighted the essential role of food quality in fostering trust. Likewise, Ladwein et al. [87] studied organic food consumers and revealed a positive effect of food quality on trust. These findings collectively suggest that food quality is a significant determinant in shaping positive consumer appraisals. Building upon this body of research, the present study proposes the following hypotheses:

**Hypothesis 1a (H1a).**  *Price fairness positively affects the trust of beef products*.

**Hypothesis 1b (H1b).**  *Price fairness positively affects the revisit intention*.

**Hypothesis 2a (H2a).**  *Freshness positively affects the trust of beef products*.

**Hypothesis 2b (H2b).**  *Freshness positively affects the revisit intention*.

**Hypothesis 3a (H3a).**  *Portion size positively affects the trust of beef products*.

**Hypothesis 3b (H3b).**  *Portion size positively affects the revisit intention*.

**Hypothesis 4a (H4a).**  *Packaging positively affects the trust of beef products*.

**Hypothesis 4b (H4b).**  *Packaging positively affects the revisit intention*.

Poon and Koay [88] identified a positive influence of trust on revisit intention among tourists in Hong Kong, while Ibrahim et al. [30] demonstrated a similar effect of trust on café revisit intention. Similarly, Thanki et al. [31] studied organic grain food consumers and found a positive relationship between food trust and loyalty. Furthermore, Sun and Moon (2024) [29] examined users of food delivery applications and revealed a positive association between trust and loyalty. It implied that trust is likely to become an essential antecedent of the repurchase intention of beef. Based on this body of literature, the present study proposes the following research hypothesis:

**Hypothesis 5 (H5).**  *Trust positively affects the revisit intention*.

### 2.5. Moderating Effect of Price Fairness Based on Heuristics

Previous research has established that price serves as a critical factor in consumer product evaluation [65,66]. Additionally, scholars have claimed that premature price discounts are likely to prompt consumers to question or suspicion to the quality of the product. Specifically, for certain consumer goods, the occurrence of price discounts may lead to doubts regarding product quality [33,89,90]. Furthermore, prior studies have indicated that when perceived price fairness is low, consumers tend to view the product as relatively expensive [8,64]. Heuristics refer to the process by which individuals make decisions based more on intuition or estimation than on rational judgment [91,92,93]. In terms of price-related aspects, previous studies documented that consumers tend to assume higher prices indicate better quality [39,40,41]. When considering this in the context of beef purchasing decisions, it implies that consumers may harbor doubts about products with lower prices, which could potentially influence their behavior. Applying these arguments to beef consumption behavior, it can be inferred that, due to the perception of beef as a premium product, a low price may trigger concerns about its quality among consumers. Moreover, heuristics can raise doubts among consumers regarding the freshness of beef when purchasing it. In other words, from the seller’s perspective, since there is a possibility that the product may have issues, the lower price could unintentionally trigger a negative response. Building on the insights from previous literature, this study attempts to explore the moderating effect of price fairness in the domain of beef consumption. Hence, the following research hypothesis is proposed:

**Hypothesis 6 (H6).**  *Price fairness significantly moderates the relationship between freshness and revisit intention*.

**Hypothesis 7 (H7).**  *Price fairness significantly moderates the relationship between trust and revisit intention*.

## 3. Method

### 3.1. Research Model and Description of Measurement Items

**Figure 2:**
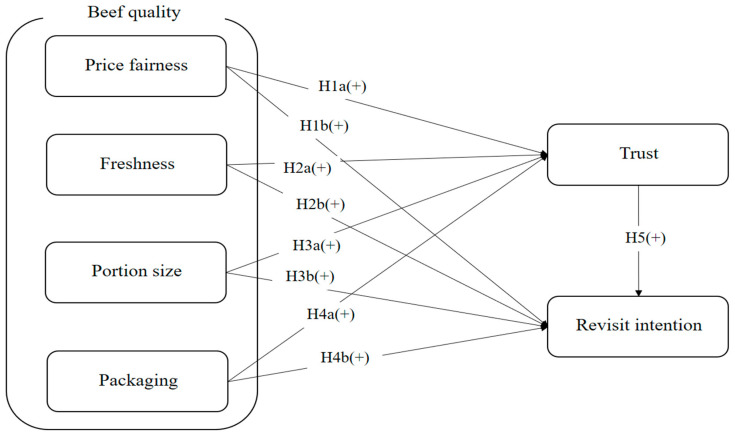
Research model.

Figure 2 illustrates the research model, which includes six attributes. The sub-dimensions of beef quality—price fairness, freshness, portion size, and packaging—are posited to positively influence trust. Furthermore, revisit intention is positively affected by price fairness, freshness, portion size, packaging, and trust.

**Figure 3:**
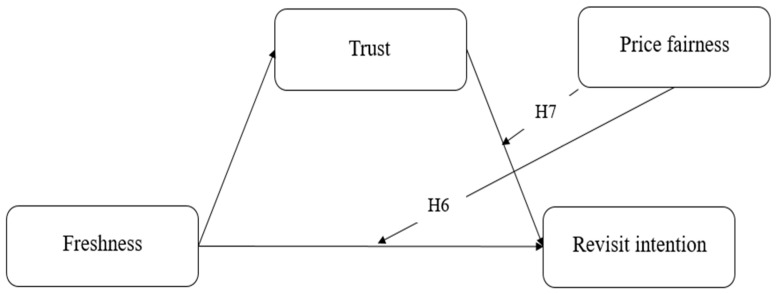
Research model focusing on the moderating effects of price fairness.

Figure 3 presents another research model, wherein price fairness is found to significantly moderate the relationship between trust and revisit intention. Also, Figure 2 presents a significant moderating role of price fairness on the impact of freshness on revisit intention.

This study measures the constructs using a five-point Likert scale (1 = Strongly Disagree, 5 = Strongly Agree). The measurement items are adapted from prior research, with necessary modifications to ensure their relevance to the present study. Table 1 presents detailed information on the measurement items along with their corresponding references. This research examines eight constructs, each consisting of four items, except revisit intention, which is measured using three items.

The operational definitions for each construct are as follows: price fairness refers to consumers’ perceptions of the price of beef products; freshness pertains to customers’ evaluations of the recentness and quality of beef production; portion size is defined as the consumer’s perception of the appropriateness of the beef serving size; packaging concerns consumers’ assessments of the wrapping and presentation of the beef products; trust reflects the degree of confidence consumers have in the quality and integrity of the beef; and revisit intention is defined as the likelihood that consumers will return to the point of sale for future beef purchases.

### 3.2. Recruitment of Survey Participants

The primary data collection instrument for this study was an online survey administered via Google’s platform. This method was selected for its flexibility, allowing participants to respond at their convenience without time or location constraints. Data collection was conducted through Clickworker (https://www.clickworker.com/), a widely recognized platform that has been successfully utilized in numerous prior studies [94,95]. Due to its reliability and broad usage, Clickworker was chosen as the primary tool for gathering responses. The survey was administered from 1 October to 7 October 2024, targeting 415 American participants. The survey aimed to assess beef consumption patterns within the U.S. market. Participants were compensated for completing the survey, which was designed to take no more than five minutes to ensure sustained engagement.

Table 2 shows the demographic information of the survey participants. The numbers of males and females are 130 and 285, respectively. Approximately 69% of participants are 30s (33.5%) and 40s (35.2%). For the weekly beef eating frequency, 1–2 times presented the largest proportion (51.8%). Table 2 additionally presents survey participants’ monthly household income information: less than USD 2500: 103, USD 2500–5000: 145, USD 5000–7500: 78, USD 7500–10,000: 24, and more than USD 10,000: 65.

### 3.3. Data Analysis

Frequency analysis was conducted to derive demographic information. A 95% confidence interval was used as the criterion for determining statistical significance. To assess the suitability of the measurement items, both convergent and discriminant validity were tested. Confirmatory factor analysis was employed to evaluate the convergent validity of the measurement items. According to previous scholars, convergent validity is confirmed using multiple indices: factor loadings greater than 0.5, average variance extracted (AVE) greater than 0.5, and construct reliability (CR) exceeding 0.7 [96,97].

Next, the means and standard deviations for the eight variables were calculated. The correlation matrix served to both examine the relationships among the variables and assess discriminant validity. Discriminant validity was evaluated by ensuring that the square root of the AVE was greater than the correlation coefficients between constructs [96,97,98]. Covariance-based structural equation modeling was employed to test the hypotheses by following the suggestion that a sample size of more than 250 is adequate to use the analytic instrument from the extant literature [96,98]. Previous works also documented that structural equation is a suitable instrument for the data analysis to examine the complex relationship between multiple attributes [96,98]. Table 3 presents the indices used to evaluate the goodness of fit for both the confirmatory factor analysis and the structural equation model, in line with established guidelines in the literature [97,98].

This research employed the ordinary least squares-based Hayes’ process model 15 using bootstrapping 5000 to test the research hypotheses. Estimation using the Hayes’ process model is less likely to be biased because the normality assumption is not mandatory in the Hayes’ process analysis [99,100]. The moderating effect was tested by generating the attributes: Freshness × Price fairness and Trust × Price fairness. This research also tested the moderating effect of freshness based on the slopes of the conditional effect of the focal predictor in the 95 percent confidence interval [99,100]. In addition, a simple slope method was employed to present the moderating effect of price fairness graphically. The simple slopes method facilitates the decomposition of interaction effects, thereby elucidating how the association between an independent variable and a dependent variable differs across varying levels of a moderating variable [99,100]. This work also inspected both cases, excluding and including control variables: gender, age, weekly eating frequency, and monthly household income.

## 4. Results

### 4.1. Confirmatory Factor Analysis and Correlation Matrix

Table 4 presents the results of the confirmatory factor analysis. The goodness-of-fit indices demonstrate the statistical significance of the model (χ^2^ = 361.162, *df* = 215, χ^2^/*df* = 1.680, RMR = 0.034, GFI = 0.929, NFI = 0.960, RFI = 0.953, IFI = 0.983, TLI = 0.980, CFI = 0.983, and RMSEA = 0.041). All factor loadings and CRs exceed the recommended thresholds of 0.5 and 0.7, respectively. Additionally, all average variances extracted (AVEs) met the criteria for validity. Table 4 also reports the means and standard deviations (SDs) for the constructs: price fairness (mean = 2.83, SD = 0.99), freshness (mean = 4.43, SD = 0.74), portion size (mean = 3.85, SD = 0.93), packaging (mean = 4.01, SD = 0.82), trust (mean = 3.60, SD = 0.96), and revisit intention (mean = 4.33, SD = 0.88).

Table 5 shows the correlation matrix. All diagonal values exceed the correlation coefficients, indicating that discriminant validity is ensured. Furthermore, revisit intention is positively correlated with price fairness (r = 0.219, *p* < 0.05), freshness (r = 0.543, *p* < 0.05), portion size (r = 0.486, *p* < 0.05), packaging (r = 0.520, *p* < 0.05), and trust (r = 0.557, *p* < 0.05). Similarly, trust is positively correlated with price fairness (r = 0.336, *p* < 0.05), freshness (r = 0.384, *p* < 0.05), portion size (r = 0.465, *p* < 0.05), and packaging (r = 0.504, *p* < 0.05).

### 4.2. Results of Hypotheses Testing

Table 6 presents the results of the hypothesis testing. The findings indicate that price fairness has a positive effect on trust (β = 0.125, *p* < 0.05). Freshness is positively associated with both trust (β = 0.129, *p* < 0.05) and revisit intention (β = 0.336, *p* < 0.05). Furthermore, portion size has positive effects on trust (β = 0.207, *p* < 0.05) and revisit intention (β = 0.126, *p* < 0.05). Packaging positively influences both trust (β = 0.326, *p* < 0.05) and revisit intention (β = 0.171, *p* < 0.05). Finally, the results confirm that trust positively affects revisit intention (β = 0.315, *p* < 0.05).

Table 7 illustrates the results of the hypothesis testing. The results of Hayes’ process macro model 15 are statistically significant based on the F-value (*p* < 0.05). Trust is positively associated with revisit intention (β = 0.936, *p* < 0.05). The interaction variable significantly affected revisit intention (β = −0.172, *p* < 0.05). Thus, H6 is supported.

**Figure 4:**
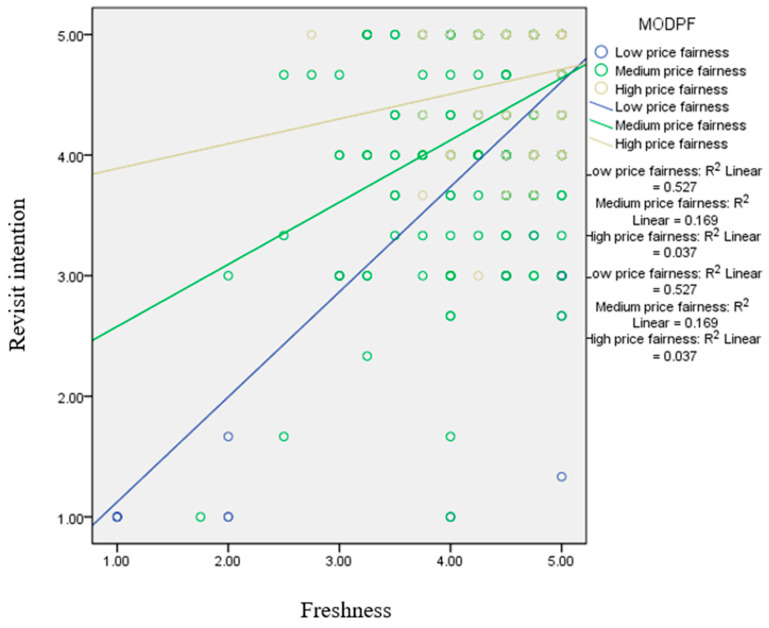
Results of the simple slope method for the moderating effect of price fairness on the relationship between freshness and revisit intention.

**Figure 5:**
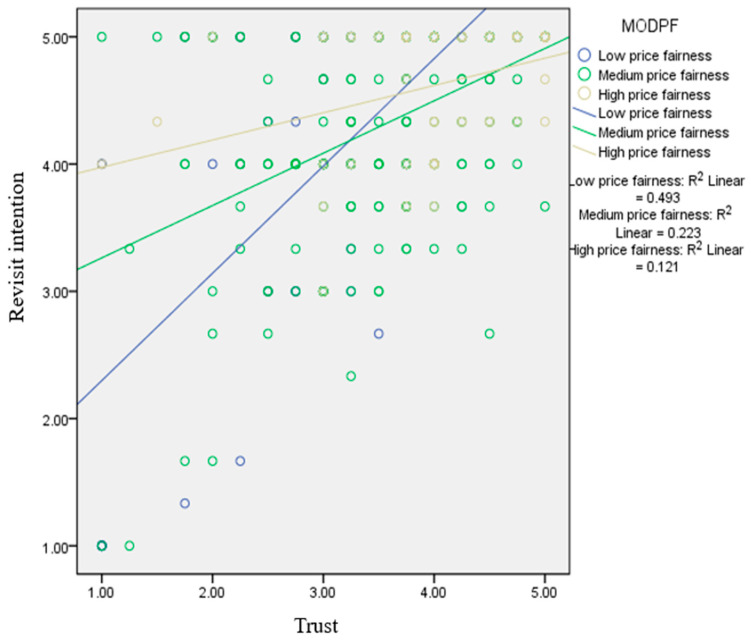
Results of the simple slope method for the moderating effect of price fairness on the relationship between trust and revisit intention.

Figure 4 shows the results of the hypothesis testing. The low price fairness group showed the highest magnitude of the effect of freshness on revisit intention (β = 0.504, *p* < 0.05). In contrast, the high price fairness group revealed a lower magnitude of the effect of freshness on revisit intention (β = 0.253, *p* < 0.05). Figure 5 exhibits the results of the simple slope method. The low price fairness group showed the highest magnitude on the influence of trust on revisit intention (β = 0.425, *p* < 0.05), while the high price fairness group exerted the lower magnitude on the effect of trust on revisit intention (β = 0.239, *p* < 0.05).

## 5. Discussion

This study investigated perceived beef quality through four key attributes: price fairness, freshness, portion size, and packaging. Among these, price fairness received the lowest mean rating (mean = 2.83), while freshness was rated highest (mean = 4.43), indicating that although consumers perceive beef as relatively expensive, they place considerable importance on freshness. The results also revealed that price fairness significantly influenced consumer trust, but did not directly impact revisit intention. This implies that while fair pricing enhances perceived credibility, it may not sufficiently offset the financial burden associated with beef, thereby limiting its impact on repurchase behavior. In contrast, freshness, portion size, and packaging all positively contributed to trust, with packaging exerting the strongest influence. Notably, appropriate portion sizes were associated with reduced food waste and alignment with dietary expectations, further reinforcing trust. Additionally, certifications (e.g., USDA Choice) on packaging enhanced perceived reliability. Freshness, portion size, packaging, and trust were all positively associated with revisit intention, with freshness emerging as the most influential factor. These results underscore the importance of freshness as a key driver of repeat patronage and highlight trust as a critical determinant of consumer loyalty in the beef sector.

The study also confirmed the moderating role of price fairness in two relationships: between freshness and revisit intention, and between trust and revisit intention. Specifically, lower prices for fresh beef were found to diminish revisit likelihood, potentially due to quality-related skepticism. Moreover, the positive effect of trust on revisit intention weakened among consumers with a higher perception of price fairness. Grounded in heuristic theory, these findings indicated that, for high-priced goods like beef, lower perceived prices may inadvertently signal lower quality, thereby attenuating the influence of trust on behavioral intentions. In other words, the research findings indicate that consumers are more likely to continue purchasing expensive food ingredients, such as beef, when a relatively high price is presented, as it conveys attributes like freshness and trust. The results also suggest that consumers tend to rely on heuristics—assuming that “expensive means better”—and make purchases based on such vague expectations. This reflects a phenomenon commonly observed with luxury goods (perfume, luxury car, luxury watch, etc.) [39,41]—namely, the belief that “expensive means better”—and suggests that consumers may perceive beef as a type of luxury food ingredient.

## 6. Conclusions

### 6.1. Theoretical Implications

This study explores four attributes—price fairness, freshness, portion size, and packaging—to better understand how consumers perceive beef quality. By examining their influence on consumer behavior, the research offers preliminary insights into the role these dimensions may play in shaping quality perceptions. The findings indicated that food quality is context-dependent, varying across consumer segments and market conditions [1,54]. In this way, the study aims to contribute to ongoing efforts to refine the conceptualization of food quality and to deepen understanding of consumer responses to beef products. This study contributes to the existing body of literature by illustrating how food quality influences both consumer trust [86,87] and revisit intention [84,85], offering valuable insights into the psychological drivers of repeat purchase behavior. Specifically, this study is significant in demonstrating that price fairness, freshness, portion size, and packaging are important factors that significantly influence consumers’ trust and revisit intention in the beef market. Furthermore, the findings contribute to theory by revealing that among these four factors, packaging exerts the strongest impact on trust, whereas freshness serves as the most influential driver of revisit intention. Moreover, this research enriches the literature by validating previous findings on the positive effect of trust on revisit intention [30,31].

Next, this study makes an important contribution by demonstrating the significant moderating effect of price fairness on the relationship between freshness, trust, and revisit intention. Such findings ensured the explanatory power of heuristics in the context of beef consumer research. Further investigation into consumer behavior within the beef product sector could deepen our understanding of these dynamics by elucidating the relationship between variables: freshness, trust, price fairness, and revisit intention. Namely, this study is worthwhile in that it sought to identify the factors that consumers consider when making purchasing decisions related to beef consumption.

### 6.2. Managerial Implications

From a managerial perspective, this study provides several key recommendations for beef product vendors. Given the importance of beef in many consumers’ diets, it is essential to maintain stable pricing strategies to avoid deterring repeat purchases. Price fluctuations may negatively affect perceptions of price fairness, which could hinder consumer trust and loyalty. Therefore, beef product managers might be able to prioritize the development of strong relationships with reliable suppliers to ensure consistent product freshness and quality. Strengthening supply chain and storage systems might also help preserve freshness, a critical factor in fostering consumer trust. Furthermore, efficient inventory turnover management is crucial, as accumulated stock may compromise product quality and freshness. Understanding market demand for portion sizes could be vital; offering flexible portion options will allow businesses to cater to diverse consumer preferences and consumption patterns, enhancing product appeal. Additionally, packaging plays a significant role in shaping consumer perceptions. Investing in high-quality, efficient packaging solutions, along with certifications such as USDA Choice and Hazard Analysis and Critical Control Points (HACCP), might be able to significantly enhance consumer trust and improve product presentation. In light of increasing environmental concerns, businesses might be able to consider using eco-friendly packaging materials to align with consumer expectations. Effective marketing communications could focus on reinforcing the product’s credibility by educating consumers on the quality and safety of the beef they purchase, thereby fostering long-term trust and loyalty. Ultimately, beef vendors might be able to emphasize relationship-building with their primary stakeholder, the consumer. By consistently delivering high-quality beef, vendors can encourage repeat visits and cultivate long-term customer loyalty. Furthermore, the findings of this study indicate that placing attention on packaging and freshness—due to their links with consumer trust and repeat purchase intention—could be considered a useful approach for more efficient resource allocation to support consumer confidence and promote continued beef consumption.

With respect to pricing, the study’s findings imply that businesses should adopt different pricing strategies based on market segmentation. Specifically, for consumers with higher purchasing power, taking a conservative approach to discounts may help nurture greater consumer loyalty. A cautious stance on discounts could be more effective in fostering long-term loyalty among these consumers, implying that discount strategies should be tailored to specific consumer segments. In other words, targeting a group that consumes high-priced beef implies that price discounts may lead to unintended outcomes, a point that can be inferred from the research findings.

### 6.3. Limitations and Directions for Future Research

This study has several limitations that warrant consideration. First, the reliance on self-reported survey data may constrain the depth of insight into actual consumer behavior. Future research could adopt more rigorous methodologies—such as experimental designs incorporating behavioral or observational data, or longitudinal approaches—to capture a more nuanced and dynamic understanding of consumer perceptions and decision-making over time. Second, the sample was predominantly female, which may introduce gender-related bias. To enhance the generalizability of the findings, future studies should aim for a more gender-balanced sample. Next, this work focused exclusively on beef—a high-priced food product—potentially limiting the applicability of the results to other, less expensive food categories. Future research could expand the scope to include other types of meat, such as chicken or pork, which may evoke different consumer expectations and behaviors due to variations in price and perceived quality. Exploring these differences could provide valuable insights into how consumer perceptions and behaviors vary across different meat categories. Furthermore, this study attempted to define beef quality from the consumer’s perspective using only four attributes. Future research might be able to consider incorporating a broader range of factors, such as safety, technological, sensory, nutritional characteristics, and convenience, or explore the definition of beef quality from the perspective of vendors or suppliers. This would offer a more understanding of beef quality from various stakeholders’ viewpoints. Finally, the sample in this study was limited to U.S. consumers, which restricts the generalizability of the findings to other geographic regions. The sample for this work primarily consisted of U.S. participants, particularly middle-aged females. However, given the cross-cultural differences in meat consumption practices and price perceptions, caution could be exercised in generalizing the findings beyond this specific demographic and cultural context. Future research could examine consumer behavior in different geographical regions, where the availability of beef and consumption cultures may vary. This would provide valuable insights into how consumer behavior related to beef differs across countries and cultural contexts, further enriching our understanding of global consumer trends.

## Figures and Tables

**Table 1:** Illustration of measurement items.

Construct	Code	Item	Reference
Price fairness	PF1	The price of beef was fair.	Singh et al. [66] Do et al. [67]
	PF2	The price of beef was reasonable.	
	PF3	The price of beef was acceptable.	
	PF4	The price of beef was affordable.	
Freshness	FR1	The color of beef was important.	Jaeger et al. [69] Lin et al. [70]
	FR2	The freshness of beef was essential.	
	FR3	For me, the freshness of beef was critical.	
	FR4	Fresh visuals of beef were imperative.	
Portion size	PS1	The portion size of beef was adequate.	Cavazza et al. [73] Petit et al. [74]
	PS2	The size of the beef was suitable for my needs.	
	PS3	The portion size of beef was appropriate.	
	PS4	The portion size of beef met my needs well.	
Packaging	PK1	The packaging of beef was adequate.	Deliya & Parmar [78] Bou-Mitri et al. [80]
	PK2	The packaging protected the beef adequately.	
	PK3	The packaging of beef was suitable.	
	PK4	The beef packaging was useful for the product.	
Trust	TR1	I trust beef.	Wu & Huang [51] Min et al. [52]
	TR2	Beef is reliable.	
	TR3	Beef is trustworthy to consume.	
	TR4	I have a credence to beef.	
Revisit intention	RI1	I intend to visit the place where I bought the beef again.	Al-Sulaiti [23] Mandagi et al. [47]
	RI2	I am going to visit the place where I purchased the beef again.	
	RI3	I will revisit the store where I purchased the beef.	

**Table 2:** Demographic information (N = 415).

Item	Frequency	Percentage
Male	130	31.3
Female	285	68.7
20s	60	14.5
30s	139	33.5
40s	146	35.2
50s	55	13.3
Older than 60	15	3.6
Weekly beef eating frequency		
Less than 1 time	71	17.1
1–2 times	215	51.8
3–6 times	116	28.0
Everyday	13	3.1
Monthly household income		
Less than USD 2500	103	24.8
USD 2500–5000	145	34.9
USD 5000–7500	78	18.8
USD 7500–10,000	24	5.8
More than USD 10,000	65	15.7

**Table 3:** Criteria of goodness of fit.

Index	Criteria
Q (chi-square/degrees of freedom)	Less than 3
Root mean square residual (RMR)	Less than 0.05
Goodness-of-fit index (GFI)	Greater than 0.9
Normed fit index (NFI)	Greater than 0.9
Relative fit index (RFI)	Greater than 0.9
Incremental fit index (IFI)	Greater than 0.9
Tucker–Lewis index (TLI)	Greater than 0.9
Comparative fit index (CFI)	Greater than 0.9
Root mean square error of approximation (RMSEA)	Less than 0.05

**Table 4:** Confirmatory factor analysis and reliability test.

Construct	Code	Loading	Mean (SD)	AVE	CR
Price fairness	PF1	0.887	2.83 (0.99)	0.798	0.940
	PF2	0.969			
	PF3	0.893			
	PF4	0.819			
Freshness	FR1	0.717	4.43 (0.74)	0.587	0.849
	FR2	0.824			
	FR3	0.845			
	FR4	0.667			
Portion size	PS1	0.887	3.85 (0.93)	0.770	0.930
	PS2	0.904			
	PS3	0.904			
	PS4	0.812			
Packaging	PK1	0.829	4.01 (0.82)	0.898	0.963
	PK2	0.893			
	PK3	0.948			
	PK4	0.823			
Trust	TR1	0.936	3.60 (0.96)	0.757	0.925
	TR2	0.924			
	TR3	0.885			
	TR4	0.719			
Revisit intention	RI1	0.939	4.33 (0.88)	0.666	0.888
	RI2	0.958			
	RI3	0.946			

Note: SD stands for standard deviation, goodness-of-fit indices: χ^2^ = 361.162, *df* = 215, χ^2^/*df* = 1.680, RMR = 0.034, GFI = 0.929, NFI = 0.960, RFI = 0.953, IFI = 0.983, TLI = 0.980, CFI = 0.983, RMSEA = 0.041, CR stands for construct reliability, and AVE is average variance extracted.

**Table 5:** Correlation matrix.

	1	2	3	4	5	6
1. Price fairness	0.893					
2. Freshness	0.115 *	0.766				
3. Portion size	0.441 *	0.439 *	0.877			
4. Packaging	0.296 *	0.421 *	0.474 *	0.947		
5. Trust	0.336 *	0.384 *	0.465 *	0.504 *	0.870	
6. Revisit intention	0.219 *	0.543 *	0.486 *	0.520 *	0.557 *	0.874

Note: * *p* < 0.05, diagonal is the square root of average variance extracted, SD stands for standard deviation.

**Table 6:** Results of hypothesis testing.

Path	Beta	t Value	*p*-Value	Results
Price fairness → Trust	0.125 *	2.534	0.011	H1a supported
Price fairness → Revisit intention	−0.031	−0.716	0.474	H1b not supported
Freshness → Trust	0.129 *	2.326	0.020	H2a supported
Freshness → Revisit intention	0.336 *	6.526	0.000	H2b supported
Portion size → Trust	0.207 *	3.483	0.000	H3a supported
Portion size → Revisit intention	0.126 *	2.403	0.016	H3b supported
Packaging → Trust	0.326 *	6.026	0.000	H4a supported
Packaging → Revisit intention	0.171 *	3.486	0.000	H4b supported
Trust → Revisit intention	0.315 *	6.590	0.000	H5 supported

Note: * *p* < 0.05.

**Table 7:** Results of the moderating effect of price fairness using Hayes Process Macro model 15.

Variables	Model 1a Beta (t Value) DV: Trust	Model 1b Beta (t Value) DV: Trust	Model 2a Beta (t Value) DV: Revisit Intention	Model 2b Beta (t Value) DV: Revisit Intention
Intecept	1.296 (4.68) *	0.844 (3.00) *	−0.926 (−2.07) *	−1.123 (−2.43)
Freshness	0.520 (8.45) *	0.395 (6.87) *	0.700 (6.06) *	0.697 (6.03) *
Trust			0.571 (5.79) *	0.558 (5.64) *
Price fairness			0.841 (4.56) *	0.841 (4.55) *
Freshness × Price fairness			−0.111 (−2.48) *	−0.115 (−2.55) *
Trust × Price fairness			−0.083 (−2.43) *	−0.079 (−2.34) *
Gender		−0.142 (−1.65)		0.199 (2.91) *
Age		0.002 (0.06)		−0.003 (−0.09)
Weekly eating frequency		0.525 (9.39) *		0.045 (0.92)
Monthly household income		−0.017 (−0.58)		0.016 (0.68)
F-value	71.49 *	36.05 *	72.16 *	41.72 *
R^2^	0.1476	0.3059	0.4687	0.4811
Conditional effect of the focal predictor Freshness × Price fairness				
1.75 (Price fairness)			0.504 (9.10) *	0.495 (8.96) *
3.00 (Price fairness)			0.365 (6.33) *	0.351 (6.07) *
4.00 (Price fairness)			0.253 (2.82) *	0.236 (2.61) *
Conditional effect of the focal predictor Trust × Price fairness				
1.75 (Price fairness)			0.425 (8.67) *	0.418 (8.21) *
3.00 (Price fairness)			0.322 (8.21) *	0.318 (7.51) *
4.00 (Price fairness)			0.239 (4.07) *	0.239 (3.89) *

Note: * *p* < 0.05, and DV denotes the dependent variable.

## Data Availability

The data presented in this study are available on request from the corresponding author. The data are not publicly available due to privacy.
